# Fluid overload and acute kidney injury: cause or consequence?

**DOI:** 10.1186/s13054-015-1163-7

**Published:** 2015-12-27

**Authors:** Marlies Ostermann, Heleen M. Oudemans-van Straaten, Lui G. Forni

**Affiliations:** Department of Critical Care and Nephrology, King’s College London, Guy’s & St Thomas’ Hospital, Westminster Bridge Road, London, SE1 7EH UK; Department of Adult Intensive Care, VU University Medical Centre, De Boelelaan 1118, 1081 HZ Amsterdam, The Netherlands; Surrey Perioperative Anaesthesia & Critical Care Collaborative Research Group Royal Surrey County Hospital NHS Foundation Trust & School of Health Sciences, Faculty of Health & Medical Sciences, University of Surrey, Guildford, GU2 7XH UK; Intensive Care Unit, Royal Surrey County Hospital NHS Foundation Trust, Egerton Road, Guildford, GU2 7XX UK

## Abstract

There is increasing evidence that fluid overload and acute kidney injury (AKI) are associated but the exact cause-effect relationship remains unclear. Wang and colleagues analysed patients admitted to 30 intensive care units in China and found that fluid accumulation was independently associated with an increased risk of AKI and mortality. This commentary focuses on the close pathophysiological link between AKI and fluid overload and discusses the implications for clinical practice. It outlines some of the challenges, including the difficulty in diagnosing fluid overload reliably with current methods, and stresses the importance of personalised fluid therapy with physiological end-points to avoid the deleterious effects of fluid overload.

Fluids are the second most common intervention in acutely ill patients (after oxygen). Whilst the benefits of early fluid resuscitation in patients with shock and acute kidney injury (AKI) have been accepted, uncertainty remains about the optimal type, volume and timing of fluid delivery. There is growing evidence that fluid administration beyond the correction of hypovolaemia is *associated* with increased morbidity, a longer hospital stay and mortality. In a recent article in *Critical Care*, Wang et al. analysed the data of 2526 patients admitted to 30 intensive care units (ICUs) in China and showed that even relatively small degrees of fluid overload were independently associated with an increased risk of AKI and mortality [1]. Others have shown associations between fluid overload and poor outcomes in patients with acute lung injury, congestive heart failure, and AKI receiving renal replacement therapy (RRT) and patients undergoing major surgery [2–6].

Although there is now little doubt that fluid overload is associated with AKI, it is difficult to disentangle the cause-effect relationship. Furthermore, translating the results into clinical practice is very challenging, especially since some important issues are still unresolved.

First, defining fluid overload is not straightforward. By convention, most studies in the literature, including the study by Wang et al. [1], defined fluid overload by a percentage increase in body weight from day of admission to the ICU. However, this assumes that patients were intravascularly replete on admission (which is often not the case). It also ignores any insensible losses as well as fluid administration in the pre-ICU setting.

Second, whether the consequences of fluid overload depend on the specific type of fluid has not been studied. It is plausible that fluid overload as a result of excessive crystalloid administration has a different impact compared with fluid accumulation following infusion of colloids or massive transfusion of blood products. More data are necessary. The timing of volume administration may also be relevant. A positive fluid balance of 5 litres over an initial 24-hour period followed by no further fluid gain may have a different outcome compared with the same net balance over a period of days.

Third, fluid overload may result from overzealous fluid administration or oliguria or a combination of the two. The differentiation is important since fluid overload caused by excessive fluid therapy is potentially avoidable whereas fluid overload as a result of oliguria may reflect AKI and may not be easily modifiable without RRT [7]. Most studies have not distinguished between the two. In a recent retrospective analysis, it was demonstrated that fluid administration, rather than low urine output, was independently associated with progression from AKI stage I to stage III [8]. Clearly, more detailed work is necessary to identify the exact factors responsible for fluid overload.

Finally, the fact that fluid overload is associated with AKI does not prove causality. One key problem is that the effects of volume overload and the effects of AKI are similar. Both lead to multi-organ dysfunction. They also often have the same denominator, including endothelial dysfunction due to inflammation or ischaemia/reperfusion injury with decay and shedding of the glycocalyx and subsequent capillary leakage [9]. Patients with more severe endothelial dysfunction tend to develop both fluid overload and AKI following fluid administration. Importantly, hypervolaemia may stretch the vascular wall and worsen vascular permeability, possibly by atrial natriuretic peptide-induced damage to the glycocalyx [10] (Fig. 1). Unpicking this vicious circle can be very difficult.

**Fig. 1:**
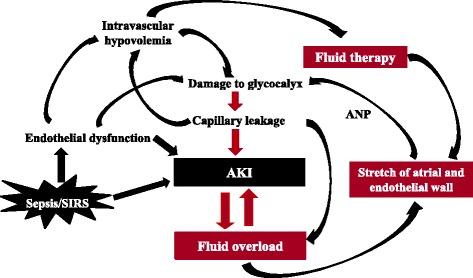
Interconnected relationship between acute kidney injury and fluid overload. *AKI* acute kidney injury, *ANP* atrial natriuretic peptide, *SIRS* systemic inflammatory response syndrome

Nevertheless, the study by Wang et al. does confirm what other studies have shown previously that a positive fluid balance is associated with worse outcomes. The crucial question for clinical practice is how to ensure appropriate and timely correction of hypovolaemia without causing fluid overload. Unfortunately, the tools to guide fluid management and to inform the clinical team when fluid administration is no longer beneficial are inadequate. Central venous pressure (CVP) or dynamic techniques like fluid responsiveness or echocardiography are often used. However, the CVP does not accurately reflect intravascular volume status and can be misleading [11]. Measuring changes in pulse pressure and stroke volume may provide very useful information for assessing preload and fluid responsiveness but does not diagnose or quantify fluid overload either. Crucially, these techniques are not readily available outside critical care areas. Cumulative fluid balance gives an indication about the fluid input–output relationship but does not correct for non-measurable losses, is often inaccurate [12] and does not indicate whether a patient is intravascularly hypovolaemic, euvolaemic or overloaded.

In the absence of any sophisticated tools to confirm euvolaemia or diagnose fluid overload, initial fluid resuscitation should be guided by an assessment of fluid responsiveness followed by fluid therapy titrated against individualized end-points [13, 14]. The passive leg raising (PLR) manoeuvre (i.e., internal fluid loading which is reversible) coupled with real-time stroke volume monitoring has been recommended to assess the need for fluid therapy [14].

There is some prospect that novel biomarkers may also have a role to prevent fluid overload. Mekontso Dessap et al. randomly assigned 304 mechanically ventilated patients to a strategy driven by B-type natriuretic peptide (BNP) or usual care [15]. Patients in the BNP-led cohort received diuretics and fluid restriction on days when their BNP was at least 200 pg/ml. Fluid balance was more negative and time to successful extubation was shorter in this group.

## Conclusions

Fluid overload, AKI and poor outcomes are clearly associated. However, whether AKI has caused fluid overload or vice versa can be difficult to determine. Better tools are necessary to measure intravascular volume status. Until then, fluid therapy needs to be personalised with physiological end-points, guided by dynamic tests like PLR manoeuvre, and monitored by using cumulative fluid balance coupled, where possible, with daily weights to avoid the adverse outcomes associated with fluid overload.

## Abbreviations

AKI: acute kidney injury
BNP: B-type natriuretic peptide
CVP: central venous pressure
ICU: intensive care unit
PLR: passive leg raising
RRT: renal replacement therapy
